# Prediction of tissue outcome in acute ischemic stroke based on single-phase CT angiography at admission

**DOI:** 10.3389/fneur.2024.1330497

**Published:** 2024-03-19

**Authors:** Frosti Palsson, Nils D. Forkert, Lukas Meyer, Gabriel Broocks, Fabian Flottmann, Máté E. Maros, Matthias Bechstein, Laurens Winkelmeier, Eckhard Schlemm, Jens Fiehler, Susanne Gellißen, Helge C. Kniep

**Affiliations:** ^1^deCODE Genetics Inc., Reykjavik, Iceland; ^2^Department of Diagnostic and Interventional Neuroradiology, University Medical Center Hamburg-Eppendorf, Hamburg, Germany; ^3^Department of Radiology, Cumming School of Medicine, University of Calgary, Calgary, AB, Canada; ^4^Department of Clinical Neurosciences, Cumming School of Medicine, University of Calgary, Calgary, AB, Canada; ^5^Alberta Children’s Hospital Research Institute, Cumming School of Medicine, University of Calgary, Calgary, AB, Canada; ^6^Hotchkiss Brain Institute, Cumming School of Medicine, University of Calgary, Calgary, AB, Canada; ^7^Department of Neurology, University Medical Centre Hamburg-Eppendorf, Hamburg, Germany

**Keywords:** stroke, infarct core, mechanical thrombectomy, deep learning, segmentation

## Abstract

**Introduction:**

In acute ischemic stroke, prediction of the tissue outcome after reperfusion can be used to identify patients that might benefit from mechanical thrombectomy (MT). The aim of this work was to develop a deep learning model that can predict the follow-up infarct location and extent exclusively based on acute single-phase computed tomography angiography (CTA) datasets. In comparison to CT perfusion (CTP), CTA imaging is more widely available, less prone to artifacts, and the established standard of care in acute stroke imaging protocols. Furthermore, recent RCTs have shown that also patients with large established infarctions benefit from MT, which might not have been selected for MT based on CTP core/penumbra mismatch analysis.

**Methods:**

All patients with acute large vessel occlusion of the anterior circulation treated at our institution between 12/2015 and 12/2020 were screened (*N* = 404) and 238 patients undergoing MT with successful reperfusion were included for final analysis. Ground truth infarct lesions were segmented on 24 h follow-up CT scans. Pre-processed CTA images were used as input for a U-Net-based convolutional neural network trained for lesion prediction, enhanced with a spatial and channel-wise squeeze-and-excitation block. Post-processing was applied to remove small predicted lesion components. The model was evaluated using a 5-fold cross-validation and a separate test set with Dice similarity coefficient (DSC) as the primary metric and average volume error as the secondary metric.

**Results:**

The mean ± standard deviation test set DSC over all folds after post-processing was 0.35 ± 0.2 and the mean test set average volume error was 11.5 mL. The performance was relatively uniform across models with the best model according to the DSC achieved a score of 0.37 ± 0.2 after post-processing and the best model in terms of average volume error yielded 3.9 mL.

**Conclusion:**

24 h follow-up infarct prediction using acute CTA imaging exclusively is feasible with DSC measures comparable to results of CTP-based algorithms reported in other studies. The proposed method might pave the way to a wider acceptance, feasibility, and applicability of follow-up infarct prediction based on artificial intelligence.

## 1 Introduction

Acute ischemic stroke is a leading cause of death and disability (1). Thrombolysis with recombinant tissue plasminogen activator (rtPA) had been the only treatment option for many years until multiple randomized controlled trials (2) confirmed high efficacy of mechanical thrombectomy (MT) in large vessel occlusions. Timely and accurate identification of the severity of the stroke with assessment of tissue infarction is critical for identifying patients that might benefit from MT. Recent advances in artificial intelligence (AI) and machine learning have led to the development of predictive models for stroke outcome using computed tomography (CT) image data and other imaging modalities (3–17). These models have shown promising results in predicting tissue infarction and the likelihood of treatment response, with potential implications for patient selection and timing of intervention (18, 19).

To date, proposed methods for tissue outcome prediction mainly utilize CT perfusion (CTP) datasets (18–20). However, accuracy of CTP-based tissue outcome prediction depends on the quality of the available CTP datasets. Factors that can influence the accuracy of CTP-based tissue outcome prediction include motion artifacts and problems related to deconvolution required to calculate the perfusion parameter maps (21). Moreover, CTP imaging is not available in all centers and not always considered a required standard of care in stroke imaging protocols.

Although perfusion imaging with assessment of core and penumbra has been established in many centers, growing evidence suggests that patient selection for MT using unenhanced CT and CTA only might also contribute to improved functional outcome. Recently published results of the TENSION RCT show that MT was associated with improved functional outcome and lower mortality in patients with established large infarct that were selected for MT based on non-contrast CT (22). These results are especially interesting as enrolled patients with large hypodense lesions at admission (ASPECTS 3–5) might not have been selected for endovascular therapy based on mismatch/perfusion although these patients benefit from MT.

Singe-phase CT angiography (CTA) is a non-invasive imaging modality that is considered standard of care in the evaluation of acute stroke patients. CTA imaging allows rapid identification of patients with large vessel occlusion (LVO) that may be amenable to endovascular treatment and provides imaging information of the collateral circulation, which is associated with patient prognosis (2). In comparison to CTP, CTA images can be acquired fast and with low technical effort and do not require costly and special licenses for acquisition and processing. Within this context, CTA datasets contain not only valuable information related to the clot location and collateral situation, but also on tissue edema formation visible as hypodense regions of the brain tissue. Despite the prognostic information available in CTA images, the value of CTA-based tissue outcome prediction using deep learning approaches has not been evaluated so far.

Thus, the goal of this work was to develop a deep learning-based algorithm that can predict the follow-up infarct location and volume based on single-phase CTA datasets only acquired acutely after patient admission. The proposed method is based on the well-established U-Net (23) architecture with several novel modifications such as residual blocks with spatial-, and channel-wise squeeze and excitation.

## 2 Materials and methods

### 2.1 Data availability

The data and code that support the findings of this study are available upon reasonable request from the corresponding author.

### 2.2 Study guidelines

The analysis was conducted in accordance with the “TRIPOD Checklist: Prediction Model Development and Validation.”

### 2.3 Study population

The study was approved by the ethics committee of the chamber of physicians at Hamburg (MC-039/16), in accordance with the Declaration of Helsinki. All patients with anterior circulation stroke due to large vessel occlusion, age ≥ 18 years, and treated at our institution with endovascular procedure between December 2015 and December 2020 were retrospectively screened. For this analysis, all patients with anterior circulation stroke and successful recanalization defined as modified Thrombolysis in Cerebral Infarction (mTICI) Scale of 2b or 3 and availability of acute CTA imaging and 24 h follow-up non-contrast CT of the brain were included. Patients with failed recanalization were excluded to eliminate effects of persistent ischemia after MT.

### 2.4 Clinical and radiologic assessment

All clinical parameters including modified Rankin Scale (mRS), vessel occlusion status and location are site reported parameters (24, 25). Reperfusion success is assessed using the mTICI scoring system (26). mTICI scoring was conducted based on the initial occlusion location and the reperfusion success within the downstream territory of the initially occluded vessel/branch. Clinical assessments and reading of baseline imaging, digital subtraction angiograms and follow-up imaging were conducted by local investigators at each participating center (single reader). Functional independence was defined as 90d mRS 0–2.

### 2.5 CT image acquisition

CT images at admission were acquired on a 2 × 128 slice scanner (SOMATOM Definition Flash, Siemens Healthcare GmbH, Erlangen, Germany) with the following imaging parameters: NCCT with 120 kV, 280 mA, less than 5.0 mm slice reconstruction and less than 0.5 mm in-plane; CTA: 100–120 kV, between 260 and 300 mA, 1.0 mm slice reconstruction, 0.5 mm collimation, 0.8 pitch, H20f soft kernel, 60 mL highly iodinated contrast medium and 30 mL NaCl flush at 4 mL/s; scan starts 6 s after bolus tracking at the level of the ascending aorta.

### 2.6 Infarct core segmentation

Manual segmentation of follow-up infarct volumes was performed using ITK-SNAP 3.8.0 (27) on 24 h follow-up CT scans (slice thickness 4.0 mm) by a senior neuroradiologist with more than 14 years of clinical experience (SG), blinded to clinical outcome data. In addition, segmentation results were visually verified by a second senior neuroradiologist with more than 20 years of clinical experience (JF). In case of disagreement, segmentations were reassessed by both readers and a consensus segmentation was generated.

### 2.7 Pre-processing

The segmentation approach uses a two-channel volume composed of the standard CTA at admission and a maximum intensity projection (MIP) as input. To eliminate uninformative rigid differences and to allow training based on the entire brain scans, input volumes were registered to a standard space CT brain atlas (28) resampled to 1x1x5 mm^3^ voxel size.

In detail, the pre-processing procedure consisted of the following steps: (1) Automatic cropping of CTA datasets to the head region using the robustFOV FSL tool (29, 30); (2) Thresholding between 0 and 400 Hounsfield units; (3) Skull stripping; (4) Deriving 5 mm MIP reconstructions of the original CTA images; (5) Registration of the non-MIP images to the CT brain standard atlas (28), application of transformation to MIP-images, registration was performed using the antsRegistrationSyNQuick command from the ANTs toolbox (31) with rigid and affine transformations; (6) Registration of the scull-stripped 24 h FU CT scan to the CT brain standard atlas using the same toolbox with rigid and affine transformations, transformation of the ground truth infarct lesion labels to standard space.

All registration outcomes were visually verified. Cases were excluded if registration failed or image quality was inadequate for evaluation.

After pre-processing, the convolutional neural network (CNN) input volume was generated by combining the registered non-MIP and MIP images into a two-channel 4D volume. The final size of this volume was 192 × 224 × 32 × 2 voxels, with spatial dimensions divisible by 24, in line with the U-Net model’s encoder stages described subsequently.

### 2.8 Model architecture

**Figure 1:**
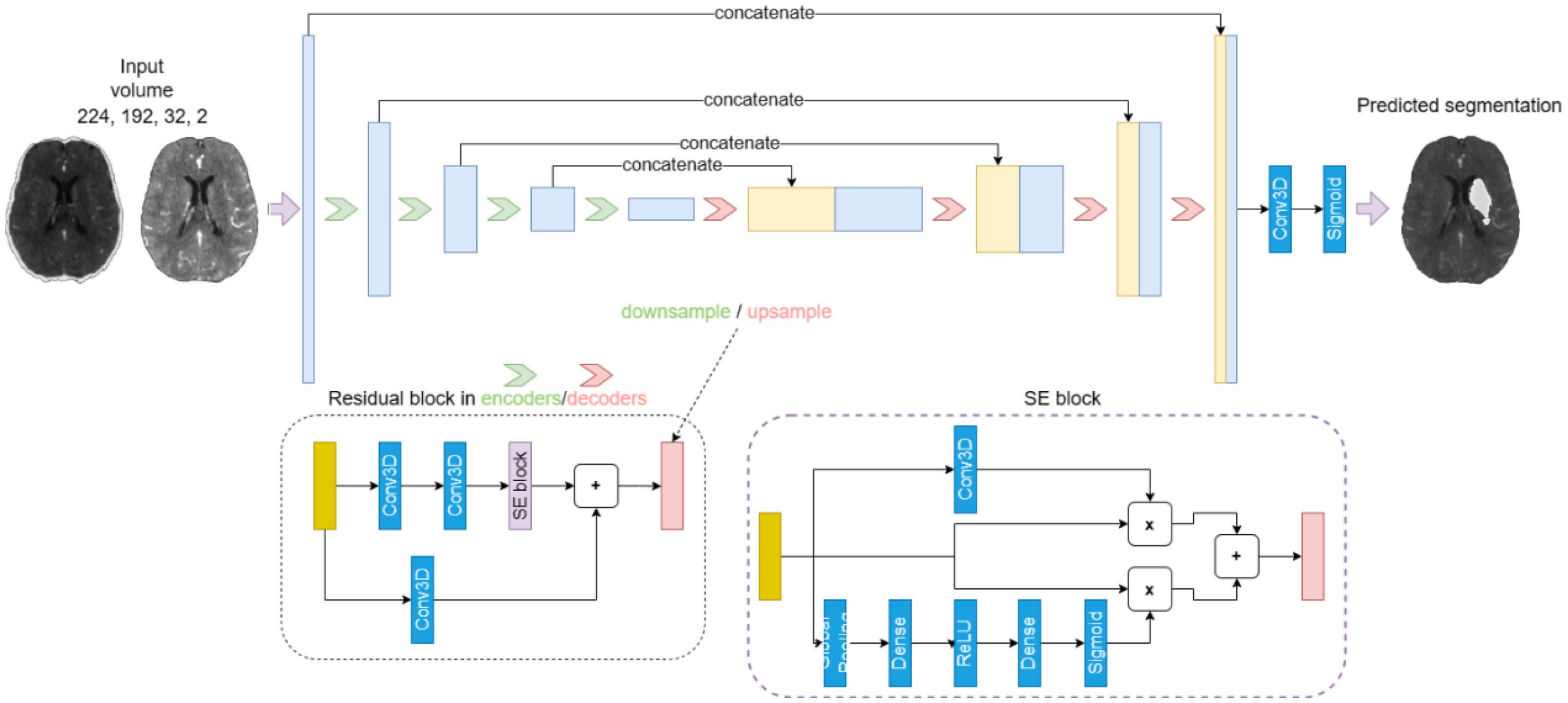
The U-Net-based 4-stage model architecture. Each stage is composed of a residual block (dotted box), which integrates two convolutional layers followed by a spatial and channel-wise squeeze and excite block (SE block, dashed box).

Figure 1 shows the architecture of our proposed lesion prediction model. It uses the well-established U-Net framework (23) as the basis, featuring a distinct encoder-decoder design with interspersed skip connections. The encoder handles feature extraction, while the decoder translates these extracted features back to the image domain. The architecture spans four stages, with each stage comprising a residual block (32). Notably, this block integrates two convolutional layers followed by a spatial and channel-wise squeeze and excite block (33) (SE block). The SE block is composed of parallel branches of spatial-and channel-wise SE blocks as shown in the lower right corner of Figure 1. The spatial part (upper branch) modulates the input feature map employing a learnable 1 × 1 × 1 convolutional layer followed by a sigmoid activation to ensure output values are scaled between [0, 1]. The weights are arranged such that each output spatial element is a linear combination of all the different channels of the input feature map for this spatial location. Therefore, the output feature map is a spatially weighted version of the input feature map, where these weights are learned during training.

The channel-wise branch modulates the weights of the separate channels of the input feature map by passing it through a global average pooling layer followed by a dense layer with an output dimension that is half the number of channels, followed by ReLU activation and another dense layer with a dimension equal to the original number of channels. Finally, a sigmoid layer ensures that the output is scaled between [0, 1]. This weight tensor is then multiplied with the input feature map to obtain an output feature map with the individual channels scaled by the learned weights. The final output of the SE block is the sum of the outputs of the spatial- and channel-wise branches.

### 2.9 Model parameters

In medical image segmentation, the region of interest is often only a small part of the total area or volume, which is also the case for tissue outcome prediction tasks. This means that the ratio of foreground voxels to background voxels can be very small and, therefore, may lead to an imbalanced problem. To address this, we used a loss function based on the Dice similarity coefficient (DSC) (34), which is a popular choice for semantic segmentation as it enables efficient training despite class imbalance.

Another important function in neural network models is the activation function, which can impact the performance and training dynamics of the model. In this work, the recently proposed Mish activation function (35) was used, which is a self-regulating, non-monotonic activation function that has been shown to improve performance compared to other popular choices such as Rectified Linear Units (ReLU).

All models were trained for a total of 300 epochs and the validation DSC was used to select the best performing model based on a check-point system. The rectified ADAM optimizer (36) with an initial learning rate of 0.001 and exponential decay with the learning rate decaying by a factor of 0.25 every 50 epochs was used.

### 2.10 Post-processing

To improve accuracy of the predicted lesion segmentation, we included a post-processing step that retained only the largest connected component of the predicted lesion. This decision is in line with the observation that most ground truth lesions consist of a single connected component. Since our model trains on entire images, its predictions predominantly reflect this characteristic. Nonetheless, occasionally, the model introduced secondary components in its predictions, which were usually inaccurate. By focusing on the largest connected component, we eliminated these erroneous predictions.

### 2.11 Experiment setup and evaluation metrics

A 5-fold nested cross-validation (CV) scheme was used for training, validation (i.e., hyper-parameter optimization) and testing. The available data (*n* = 238) was randomly split into five training/validation sets (80%, *n* = 191) and five separate test sets (20%, *n* = 47). Each training/validation set was again randomly split into 5 training sets (80% of each outer CV training/validation set, *n* = 152) and 5 validation sets (20% of each outer CV training/validation set, *n* = 39) for hyperparameter tuning. Random splits were conducted using a stratified approach based on the lesion size. This ensures a similar lesion size distribution in both sets. For each outer CV run (models 1 to 5) the average DSC of the corresponding test set was used as the primary evaluation metric while the average absolute lesion volume difference of the corresponding test set was used as a secondary evaluation metric.

### 2.12 Multivariable regression analysis

The association of segmented vs. predicted volumes with functional independence (90 day mRS 0–2) was analyzed using multivariable logistic regression. For initial neurological status (NIHSS at admission), a linearized association was assumed. Regression models were adjusted for age and pre-stroke mRS, adjusted odds ratios (aOR), coefficients, 95% confidence intervals and *p*-values were reported. p-values <0.05 were defined as statistically significant. Regression analysis was conducted with Stata/MP 18.0.

## 3 Results

**Figure 2:**
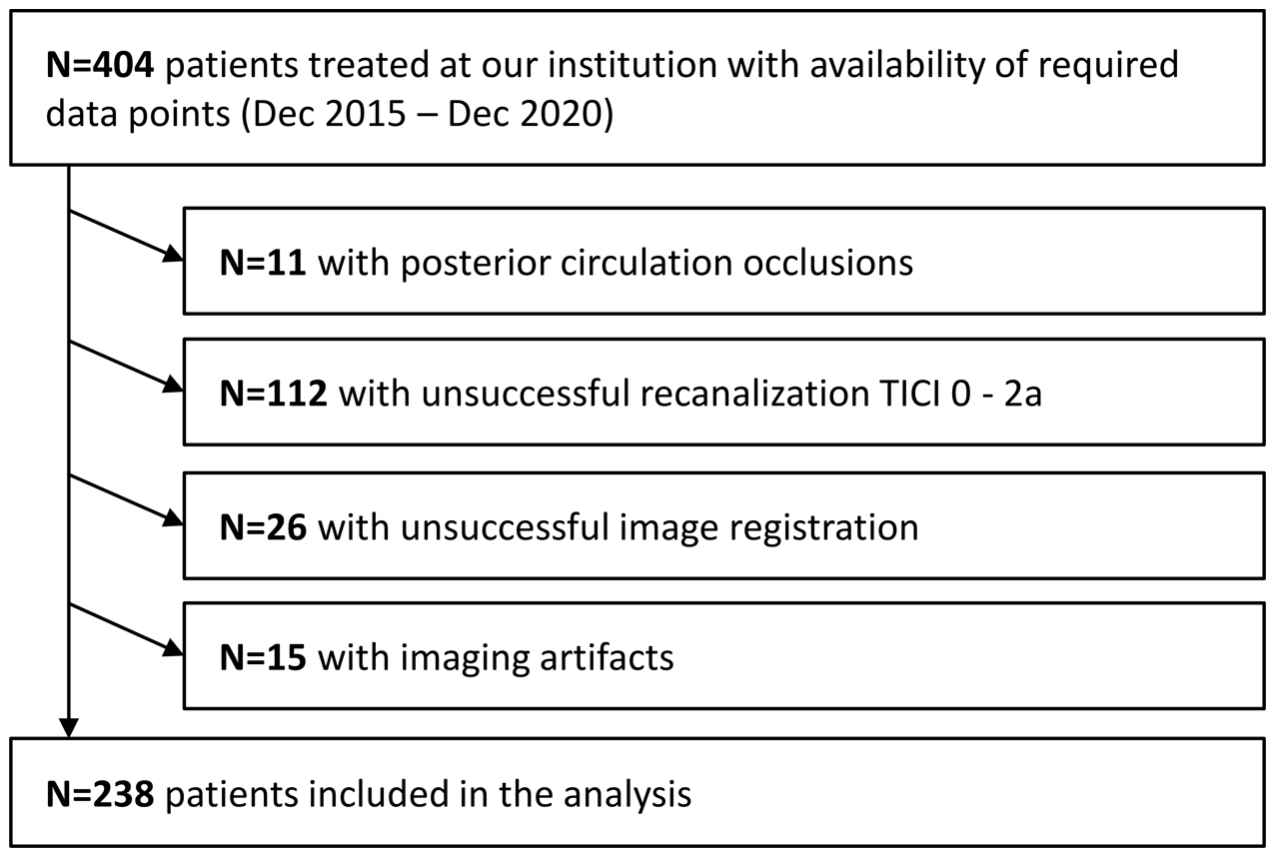
Patient inclusion flowchart.

**Figure 3:**
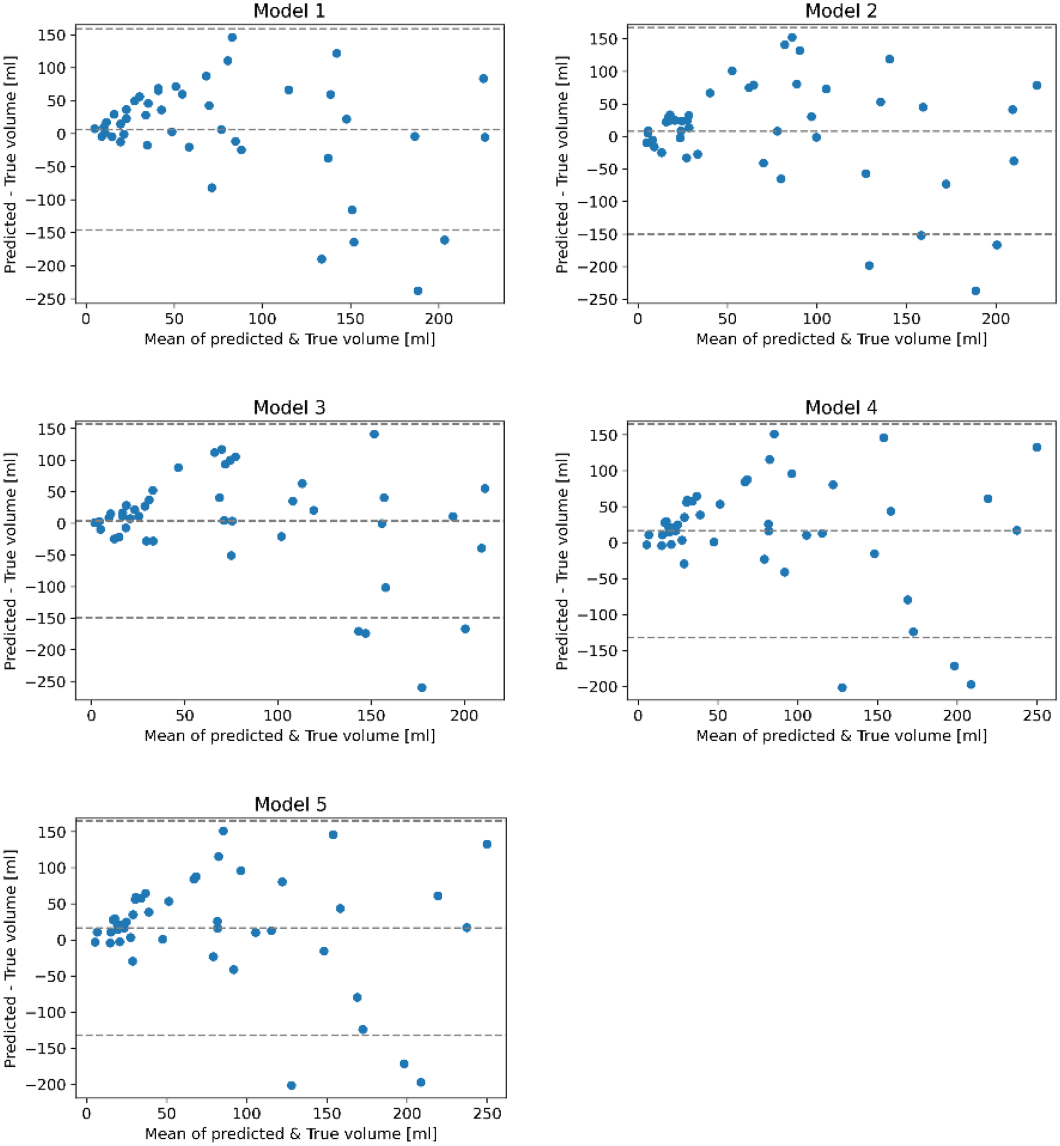
Bland–Altman plots comparing the true and predicted lesion volumes in ml for trained models on each loop of the 5-fold cross-validation after applying post-processing. Each plot is a scatterplot of the difference of the true and predicted volume vs. the mean of the predicted and true volume. The mean bias and regression based 95% limits of agreement are shown using dotted horizontal lines.

A total of 404 patients were screened, and 238 patients were included in the analysis (Figure 2). Included patients had a median age of 76 years (IQR: 64; 81), a median NIHSS at admission of 16 (IQR: 11; 19), median ASPECTS of 7 (IQR: 6; 9), median infarct volume of 25 mL (IQR: 8; 114) and median 90-days mRS of 4 (IQR: 1; 5; Table 1). Table 2 displays the test performance of the five cross-validation models, including DSC and volume error (ml) for each outer fold test set. The results are presented without and with post-processing. Figure 3 shows Bland–Altman plots comparing the true vs. the predicted lesion volumes in ml for each model.

**Table 1:** Baseline clinical characteristics of study cohort.

Variable	Median | n (%)	Q1; Q3	Range
Age (median)	76	64; 81	29–97
Sex (f)	121 (51%)		
Pre-stroke mRS (median)	0	0; 1	0–5
NIHSS at admission (median)	16	11; 19	0–42
Comorbidity hypertonus	158 (66%)		
Comorbidity diabetes	39 (16%)		
Comorbidity dyslipidemia	33 (14%)		
Comorbidity atrial fibrillation	88 (37%)		
ASPECTS at admission (median)	7	6; 9	1–10
i.v. thrombolysis	135 (57%)		
# of passes (median)	2	1; 2	0–8
AE vasospasm	5 (2%)		
AE clot migration/embolization	6 (3%)		
AE dissection/perforation	3 (1%)		
AE ICH	3 (1%)		
Final TICI			
-2b	115 (48%)		
−3	123 (52%)		
Follow-up infarct volume 24 h CT (ml)	24.8	8.3; 114.2	0–516.1
90-days mRS (median)	4	1; 5	0–6

AE, Adverse event; ASPECTS, Alberta Stroke Program Early CT Score; ICH, Intracranial hemorrhage; mRS, modified Rankin Scale; NIHSS, National Institute of Health Stroke Scale; Q1, 1st quartile; Q3, 3rd quartile; TICI, Thrombolysis in cerebral infarction.

**Table 2:** Test set results as mean ± standard deviation for each outer loop of the 5-fold cross-validation models without and with post-processing.

Without post-processing			With post-processing	
Model	DSC	Volume Error [ml]	DSC	Volume Error [ml]
1	0.36 ± 0.2	11.4 ± 79	0.37 ± 0.2	6.2 ± 79
2	0.33 ± 0.2	13.5 ± 83	0.33 ± 0.2	8.4 ± 83
3	0.34 ± 0.2	29.6 ± 78	0.34 ± 0.2	22.6 ± 78
4	0.34 ± 0.2	9.9 ± 78	0.34 ± 0.2	3.9 ± 80
5	0.35 ± 0.2	25.1 ± 77	0.36 ± 0.2	16.3 ± 77
Mean	0.34 ± 0.2	17.9 ± 79	0.35 ± 0.2	11.5 ± 79.4

DSC, Dice similarity coefficient.

Results suggest that all models perform similarly with rather small quantitative differences regarding the test set metrics with average DSC values ranging between 0.33 and 0.37 (SD: ±0.20). Likewise, the standard deviation of the DSC is similar for all models. Post-processing only slightly improved the average DSC but considerably reduced the average volume error (11.5 mL (SD: ±79.4 mL) after post-processing).

Bland–Altman plots in Figure 3 revealed that all models tend to overestimate the true infarct volume, which was also supported by the volume error (2). Furthermore, data indicated a trend for underestimating the volume of very large infarcts.

**Figure 4:**
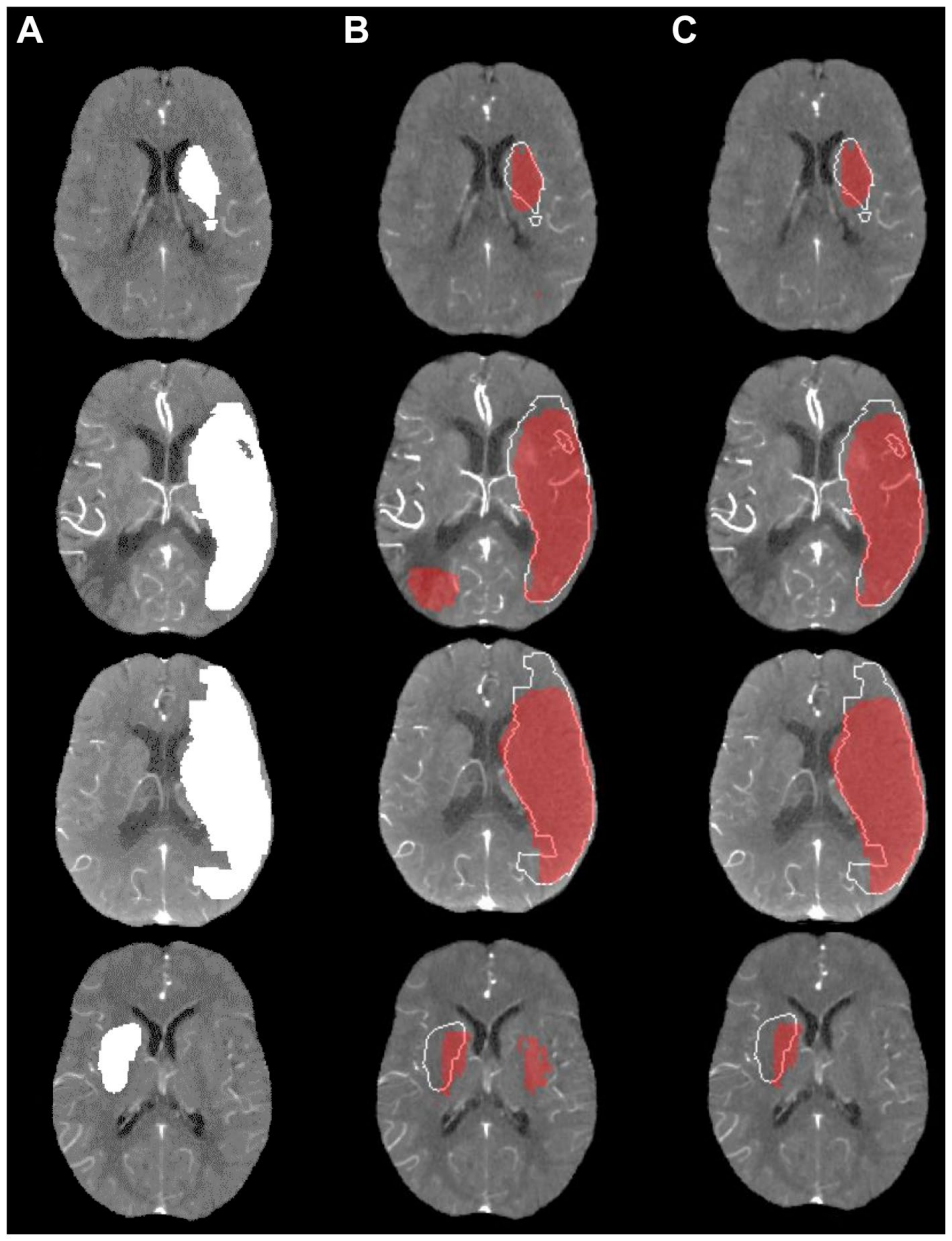
Visualization of lesion outcome prediction results obtained using the best model in terms of the average test set dice similarity coefficient for four exemplified patients. **(A)** Ground truth; **(B)** Prediction including small components red, ground truth white; **(C)** Prediction excluding small components red, ground truth white.

Figure 4 shows a visualization of the results obtained using the model with the highest average DSC compared to the ground truth for four exemplified patients. In these examples, the lesion was correctly located in all cases. However, the shape of predictions and ground truth do not fully align. It can be also seen that post-processing was primarily helpful to remove false lesions, especially those located in the contralateral hemisphere.

Table 3 shows the association of segmented in comparison to predicted volumes with initial neurological status (NIHSS at admission) and functional independence (90 day mRS 0–2). Results suggest that higher follow-up infarct volumes are associated with lower probability of functional independence at day 90 for segmented volumes (aOR: 0.98 [95% CI, 0.97–1.00], *p* < 0.05) and predicted volumes (aOR: 0.98 [95% CI, 0.96–0.99], *p* < 0.01). Both coefficients were not statistically significantly different. For NIHSS at admission a correlation with the predicted volume (Coeff: 0.07 [95% CI, 0.04–0.09], *p* < 0.001) was observed, however, for segmented volumes, the coefficient was not statistically significant (*p* = 0.112).

**Table 3:** **(A)** Multivariable logistic regression with functional independence (mRS 0–2 at 90 days) as dependent variable; **(B)** Multivariable linear regression with NIHSS at admission as dependent variable.

A: 90 days mRS 0–2 (Multivariable logistic regression)								
	Segmented volumes				Predicted volumes			
	aOR	*p* value	95% Conf. interval		aOR	*p* value	95% Conf. interval	
Volume (ml)	0.98	0.013	0.97	1.00	0.98	0.003	0.96	0.99
Age (years)	0.94	0.064	0.89	1.00	0.94	0.059	0.88	1.00
Pre-stroke mRS	0.82	0.681	0.33	2.07	0.96	0.939	0.36	2.54
Constant	109.89	0.025	1.78	6,780	376.34	0.011	3.87	36,605
B: NIHSS admission (Multivariable linear regression)								
	Segmented volumes				Predicted volumes			
	Coeff	P > t	95% Conf. interval		Coeff	p-value	95% Conf. interval	
Volume (ml)	0.02	0.112	0.00	0.04	0.07	<0.001	0.04	0.09
Age (years)	−0.07	0.373	−0.21	0.08	−0.06	0.321	−0.18	0.06
Pre-stroke mRS	0.13	0.906	−2.08	2.34	−0.78	0.398	−2.63	1.07
Constant	18.97	<0.001	9.33	28.62	14.93	0.001	6.85	23.01

mRS, Modified Rankin Scale; NIHSS, National Institutes of Health Stroke Scale; aOR, Adjusted odds ratio; Coeff, Coefficient; CI, Confidence interval.

## 4 Discussion

In this analysis, we sought to evaluate if CTA datasets can be used for tissue outcome prediction in patients with ischemic stroke. The most important finding of this work is that a prediction of tissue outcome using exclusively CTA datasets is generally feasible using deep convolutional neural networks. Test set performance of our models reached an average DSC of 0.35 with mean volume error of 11.5 mL.

Overall, the predictive performance achieved in this work is within the range of previously described tissue outcome prediction models using more complex 4D CT perfusion datasets. For example, Amador et al. achieved an average DSC of 0.45 using a very advanced temporal convolutional neural network and 4D CTP datasets as input (20), while Qiu et al. achieved mean volume error of 21.7 mL using multiphase CTA images (4). It might be argued that a deep learning model using a single time-point CTA cannot outperform an advanced method having access to the complete hemodynamic perfusion information from a 4D CTP scan. However, CTP datasets typically used for lesion outcome prediction are often highly curated by excluding datasets with severe motion or other artifacts, which they are very sensitive to. This is one of the main benefits of the here proposed method, which uses simple single time-point CTA images that are less prone to motion and other artifacts and are widely available without any costly licenses for acquisition and processing.

Another benefit of the proposed method is that the input images are used en bloc in the model instead of splitting them into smaller patches during training and inference. In this way, the network sees the whole image at once, which might result in better learning of typical infarct locations and lesion distributions with regards to the entire brain. Within this context, better lesion outcome predictions may be possible with multi-phase CTA images or by combining CTA and CTP images (5). However, this would also require a more complex deep learning model that is capable of making use of the temporal information available in multi-phase CTA images and CTP images. Also, this approach would be more prone to movement artifacts and require more complex data leading to decreased feasibility and generalizability.

The quantitative results show that the proposed model leads to an underestimation of large lesions and overestimation of small lesions. This is a common problem of many segmentation methods (regression to the mean (37)). A potential solution to this problem may be a modification of the loss function to also include the volume error or to train multiple models for different lesion sizes (6). However, the second option would also reduce the number of datasets available for training of the lesion prediction model.

Furthermore, an overestimation of infarct volume in CTA source images has also been noted in previous studies (7) where it is postulated that a possible reason for this is that modern rapid-acquisition CT scanners may produce CTA images that are more strongly CBF- than CBV weighted (38), and therefore overestimate the true infarct volume. For example, in a study of 105 patients (8), it was found that follow-up infarct volume predictions based on CTA source images significantly overestimated the infarct size in many cases.

In line with results from previous studies, multivariable regression analysis suggest that follow-up infarct volume is associated with functional outcome (39–41). Furthermore, no statistically significant difference in association with functional outcome was observed for ground-truth follow-up volume segmentations and model predictions, suggesting that predicted infarct volumes might serve as additional surrogate marker for functional outcome. For NIHSS at admission a significant association with predicted infarct volumes was observed, however, segmented volumes were not significantly associated with NIHSS at admission. One explanation could be that predicted volumes are derived from CTA imaging at admission and might therefore better reflect neurological status at admission.

This work has multiple limitations that should be discussed. First, no comparison to CTP-based lesion outcome prediction methods was conducted as corresponding CTP datasets were not available for all patients. Also, no comparison to non-enhanced CT-based methods was conducted. However, it can be assumed that tissue density information from non-enhanced CT is also included in CTA scans. Second, the proposed method did not include any clinical parameters in the model although they may improve the prediction accuracy. To date, there is no consensus regarding the optimal way to integrate clinical information in segmentation methods. Therefore, we restricted the proposed model to imaging information only to test the general feasibility of using only CTA datasets for this purpose. Third, the proposed method was only trained and tested using datasets from patients with a large vessel occlusion of the anterior circulation treated with MT. Thus, it remains unclear how well the proposed method can predict lesion outcomes in patients with an occlusion in the posterior circulation. Fourth, ground truth segmentation of the infarct core was conducted manually based on 24 h follow-up CT images. Interrater variability of manual segmentation processes might reduce the generalizability of results. However, the conducted visual verification of segmentations by a second reader and reassessment in case of disagreement increased validity of segmentation results. Fifth, model training and testing was conducted based on single-center data. However, besides external testing, the conducted nested cross validation is currently considered as gold standard for machine learning approaches. Although model training and testing was conducted based on single center data, it can be argued that CT imaging data of current state-of-the-art CT scanners has a high degree of standardization. Even if a certain center-specific bias cannot be excluded, it needs to be discussed if center-specific training and models on the other hand might allow higher predictive performance. In fact, several FDA-approved machine learning-based tools for acute stroke diagnostics require center-specific training of their algorithms. Sixth, the CNN was trained using 4D volumes generated from CTA images and MIP reconstruction resampled to 1 mm x 1 mm x 5 mm voxel size. Resampling to 5 mm slice thickness might reduce predictive precision especially for small infarcts.

## 5 Conclusion

24 h follow-up infarct prediction exclusively using acute single-phase CTA datasets is feasible and can be successfully achieved with good accuracy. In comparison to CTP data, CTA data is technically more widely available, in general incorporated into standard basic acute stroke protocols in clinical routine practice, and less prone to movement artifacts. The method proposed in this work based on single-phase CTA might pave the way to a wider acceptance, feasibility, and applicability of follow-up infarct prediction employing artificial intelligence methods.

## Data availability statement

The raw data supporting the conclusions of this article will be made available by the authors, without undue reservation.

## Ethics statement

The studies involving humans were approved by Ethics committee of the chamber of physicians at Hamburg. The studies were conducted in accordance with the local legislation and institutional requirements. Written informed consent for participation was not required from the participants or the participants’ legal guardians/next of kin in accordance with the national legislation and institutional requirements.

## Author contributions

FP: Conceptualization, Data curation, Formal analysis, Methodology¸ Software, Validation¸ Visualization, Writing – original draft. NF: Conceptualization, Data curation, Formal analysis, Supervision, Validation, Visualization¸ Writing – original draft. LM: Investigation, Project administration, Validation, Writing – review & editing. GB: Formal analysis, Investigation, Supervision, Validation, Writing – review & editing. FF: Conceptualization, Data curation, Project administration, Supervision, Validation, Writing – review & editing. MM: Project administration, Supervision, Validation, Writing – review & editing. MB: Conceptualization, Project administration, Supervision, Validation, Writing – review & editing. LW: Software, Supervision, Validation, Writing – review & editing. ES: Project administration, Supervision, Validation, Writing – review & editing. JF: Methodology, Project administration, Supervision, Validation, Writing – review & editing. SG: Conceptualization, Formal analysis, Methodology, Project administration, Supervision, Validation, Writing – original draft. HK: Conceptualization, Methodology, Project administration, Supervision, Validation, Writing – original draft.

## Glossary

AE: Adverse event
AI: Artificial intelligence
aOR: Adjusted odds ratio
ASPECTS: Alberta Stroke Program Early CT Score
CI: Confidence interval
CNN: Convolutional neural network
Coeff: Coefficient
CTA: Computed Tomography Angiography
CTP: Computed Tomography Perfusion
CV: Cross validation
DSC: Dice similarity coefficient
ICH: Intracranial hemorrhage
LVO: Large vessel occlusion
mRS: Modified Rankin scale
MIP: Maximum intensity projection
MT: Mechanical thrombectomy
NIHSS: National Institutes of Health Stroke Scale
Q1: 1st quartile
Q3: 3rd quartile
rtPA: recombinant tissue plasminogen activator
SD: Standard deviation
SE Block: Squeeze and excite block
sICH: Symptomatic intracranial hemorrhage
mTICI: modified Thrombolysis in cerebral infarction scale
